# Metacognition and abstract concepts

**DOI:** 10.1098/rstb.2017.0133

**Published:** 2018-06-18

**Authors:** Nicholas Shea

**Affiliations:** 1 Institute of Philosophy, School of Advanced Study, University of London, London WC1E 7HU, UK; 2 Faculty of Philosophy, University of Oxford, Oxford OX2 6GG, UK

**Keywords:** abstract concepts, grounding, linguistic labels, deference, metacognition, philosophy

## Abstract

The problem of how concepts can refer to or be about the non-mental world is particularly puzzling for abstract concepts. There is growing evidence that many characteristics beyond the perceptual are involved in grounding different kinds of abstract concept. A resource that has been suggested, but little explored, is introspection. This paper develops that suggestion by focusing specifically on metacognition—on the thoughts and feelings that thinkers have about a concept. One example of metacognition about concepts is the judgement that we should defer to others in how a given concept is used. Another example is our internal assessment of which concepts are dependable and useful, and which less so. Metacognition of this kind may be especially important for grounding abstract concepts.

This article is part of the theme issue ‘Varieties of abstract concepts: development, use and representation in the brain’.

## 1. Which grounding problem?

This special issue asks how abstract concepts are *grounded*—grounded not just in perception and action, but also in language, sociality, emotions, interoception and introspection. I want to focus on metacognition, which falls broadly within the last category, but first we need to look at what the grounding problem is. I distinguish two different kinds of grounding problem.

The first is the problem of how a mental item can refer to or be about the non-mental world at all. Thoughts and concepts are in the mind, yet they manage to reach out and be about things in the world. How does my concept fairness manage to pick out and concern *fairness*;^1^ or indeed how does my concept cat manage to be about cats? I will call this the problem of the metaphysics of reference: what makes it the case that a mental state or process refers to the object or property in the world that it does, in fact, refer to?

The problem of the metaphysics of reference is a perfectly general problem. It applies with equal force to perceptual states. How does my visual experience of a green cube manage to concern the shape and colour of an object? How does my visual memory of a sunset manage to represent the shapes and colours of the round red disc of the sun and its myriad reflections off the shimmering surface of the sea? For example, when Lupyan & Winter [1] argue that abstraction may sometimes be achieved by highlighting one dimension of a multifaceted icon, that effectively assumes that the relevant dimension is already represented in perception. Perceptual states are so immediate that it is hard to see that there is a problem here at all. Of course, they are about objects and properties in your environment. But: how so? If we are concerned with the general question of how mental states can be about the world, then perceptual experiences pose the very same problem. We cannot just help ourselves to the aboutness of perceptual experiences.

A theory of reference takes some characteristics of a concept as input and delivers a referent as output. A theory that said use fixes reference would say that, because concept C is used in such-and-such ways, it refers to *C*s. Various characteristics of a concept are candidates here—not just the circumstances in which C is applied to things in the world, but also other mental states connected to C and the downstream effects of applying C: the inferences that are drawn and the actions produced or potentiated. The characteristics of a concept that a theory of the metaphysics reference takes as input can be thought of as grounding the concept. If two concepts C and D are to refer to different things, then they must have different *grounding characteristics*. They must differ in the characteristics that the theory of the metaphysics of reference takes as input. (Which characteristics these are depends upon which theory of reference is the right one.)

The second grounding problem is to say what the grounding characteristics of a concept C are that are distinctive of C. Which characteristics, when input into the correct theory of reference, get C to refer to *C*s rather than other referents? This *distinctive grounding characteristics problem* will be my focus here. We are seeking characteristics that are distinctive from the point of view of the theorist of reference. They need not be the way the thinker herself distinguishes between concepts. Indeed, characteristics that correctly characterize some uses of a concept need not be apparent to the concept user (during other uses of the concept, or at all). So ours is not the cognitive significance problem: the need for an account of the cognitive difference, for the thinker, between two concepts. Nor is it the detector problem, the problem of saying how a person manages to pick out the things in the world that fall under C. Both these latter concern how the concept user succeeds in distinguishing—between concepts (in the first case) or between classes of things in the world (in the second case). Our problem is rather to say which characteristics from a metaphysical point of view ground reference. To do so differently for different concepts, those grounding characteristics must be distinctive.

The distinctive grounding characteristics problem arises in a special way for abstract concepts. Concrete concepts like cat and round are plausibly grounded in sensory experiences like the sight, sound and feel of cats, and probably also in motor experiences like the motor preparation for stroking a cat or grasping a round object. The set of sensory prototypes or exemplars associated with my cat concept, and its action affordances, plausibly ground reference to a different category than the characteristics associated with my dog concept do. For concepts like electron and fairness, it is much less plausible that the sensorimotor furnishes grounding characteristics that are sufficiently rich to be distinctive. The concepts that have been labelled as ‘abstract’ cover a wide and seemingly heterogeneous range, but they share this feature. They seem to call for distinctive grounding characteristics beyond the sensorimotor.

The grounding problem for abstract concepts is sometimes considered especially troublesome for embodied accounts of concepts [2,3]. However, those who view concepts as amodal symbols also have to face the distinctive grounding characteristics problem ([4] makes a related point). Just postulating different amodal symbols for fear and anger does not yet answer the question of what is distinctive of one vis-à-vis the other so as to be the basis of reference determination. There has to be something about the way thinkers apply and use the concept fear that makes it distinctive from the concept anger—some features that a theory of reference can hook onto to deliver the result that fear picks out instances of *fear* and anger *anger*. Having an amodal symbol does not magically make that problem disappear. The resources that embodied theories of concepts will rely on to ground abstract concepts are also likely to be needed by theories that take concepts to be amodal symbols.

## 2. Resources beyond the sensorimotor

As this special issue shows, there is a wide range of resources beyond the sensorimotor that can ground abstract concepts. The label ‘abstract concepts’ covers a large diversity of domains. Different resources are relevant for different domains. For example, for numerical concepts the capacity for tracking analogue magnitudes [5], shared with non-human animals, is important. Although not paradigmatically perceptual, this capacity can be considered quasi-perceptual in that it acts in a fast, automatic way on domain-specific input [6]. Being linked to different analogue magnitudes will help to distinguish one number concept from another, but is unlikely to be enough on its own, because the analogue magnitude system tracks numerosity only approximately.

Susan Carey's account of the acquisition of natural number concepts relies on another non-conceptual system, the object file system for tracking small numbers of objects [7]. Her sophisticated theory shows how the grasp of numerosity implicit in the object file system can be combined with linguistic resources (count words, plural markers) to produce symbols for natural numbers. These symbols could be amodal, or equally could be sensorimotor symbols based on the count words. These symbols are in turn integrated with the analogue magnitude system (which is not modality-specific). This is an empirically well-supported answer to the distinctive grounding characteristics problem, one that relies on linguistic and numerosity-based resources beyond the paradigmatically perceptual. As such, it offers a template for answering the distinctive grounding characteristics problem for abstract concepts.

With other kinds of abstract concepts, other sources of grounding are called for. For emotion concepts like fear and anger, the thinker's own emotional experiences are likely to be important. Indeed, neural evidence suggests that abstract concepts generally involve more affective processing than concrete concepts [8]. Relatedly, Wiemer-Hastings & Xu [9] found, in a property generation task, that abstract concepts were associated with more introspective and situational features than concrete concepts.

How does the affective processing involved in using an abstract concept help ground the concept? Hedonic valence is an important dimension of variation (good/bad, and the strength of valence). Abstract concepts vary in affective valence, and strength of valence explains why subjects are faster in recognizing abstract words in a lexical decision task, once imageability is controlled for [8]. Neural areas for emotion processing that track these valence differences [10] mark a distinction between abstract concepts. Part of what differentiates hope from pity, say, may be the differently valenced affective experiences engendered in the thinker when they use these different concepts. However, valence alone is insufficiently discriminating. It is unlikely to distinguish between happy and content, for example. Even if we add in arousal (although that was not found to produce significant differences in neural activation by Vigliocco *et al*. [10]), the two dimensions are unlikely to differentiate between all emotion concepts, e.g. anger and fear, or being content and being calm [11]. We also need to distinguish between emotion concepts and concepts of non-emotional mental states, e.g. hope versus want.

Within the sensorimotor, the link between a concept and action should not be overlooked. Different emotions are linked to different emotion-expressing actions [12]. We talk of ‘fight *or* flight’, and an angry person may indeed prepare a different suite of actions than a frightened one. If an emotion concept is linked to the types of action the agent will typically prepare when experiencing that emotion [13], or indeed to the types of action expected in others, then that will help to differentiate between emotion concepts.

These resources are doubtless important, but many doubt whether they can capture all the differences between our concepts. Even if affect and action are sufficient for emotions, the case is harder for concepts of other mental states, e.g. distinguishing hope from desire, or desire from intention. These are cases where the structure of the concepts seems to be important, for example as expressed in contingencies like ‘if … then …’ and ‘… because … ’ [9]. When we expand our focus to other domains of abstract concepts, it becomes even clearer that affectivity, while important, cannot be a full answer to the distinctive grounding characteristics problem. Consider moral categories like ingratitude, aesthetic categories like beauty and social categories like emancipation. Their distinctive characteristics are likely to go beyond the sensorimotor and the affective. For these reasons, many have thought that language must be important for grounding abstract concepts (see, for example, the theories surveyed in Borghi *et al*. [2]).

## 3. Linguistic resources

Words are a rich resource for making one concept distinct from another. anger is linked to a different word from fear. Being linked to different words is a factor that differentiates between concepts. Words are not, however, by themselves enough to solve the distinctive grounding characteristics problem. Words do not magically carry meanings with them. Rather, words are invested with meaning because of the concepts people use them to express (perhaps also because of the concepts people use when understanding the words they hear). Whether words are represented amodally or in a sensorimotor format, they are simply symbols to which meanings can attach. The fact that anger and fear are attached to different words is not a distinctive characteristic on which reference to different emotions can be founded. Just having the word ‘anger’ does not by itself give the thinker a way of identifying instances of anger, drawing appropriate inferences or acting appropriately when they do. The story about linguistic resources needs to be supplemented before it can help to address the distinctive grounding characteristics problem.

Words can help in two ways, one internal and the other external to the thinker. Starting in this section with the internal, words are a means by which a thinker acquires or exercises the capacities needed to have a concept. For example, in Carey's account of natural number concepts discussed above [7], part of what grounds the concept six is that, if you add one object to a collection of six objects, you get a collection that falls under the concept seven. That is an inferential disposition within an individual thinker. Inferential dispositions are resources that can help to make distinctions between different concepts. To understand how the thinker came to have that inferential disposition developmentally, according to Carey we need to look at the role of language. By learning the sequence of count words (‘six’ and ‘seven’) by rote, as uninterpreted sounds, the thinker internalizes a sequence that they can line up with the cardinal ordering of natural numbers. Words in the public language are a developmental means for acquiring the structures that differentiate between different concepts; and then linguistic labels are called on when the thinker exercises that capacity (has a thought using the concept).

Language is likely to be important for learning mental state concepts. Although babies and young children are able to track what others have seen [14], and to some extent what agents are likely to do on the basis of what they have seen (but without being able to track object identity: [15]), it is not until about 4 years of age that children give accurate answers to questions about how people will behave in false belief situations [16]. The concept of belief that children are using at this stage probably depends on language [17]. The same may be true of logical concepts. Even with a concept as simple as or, children make surprising reasoning errors until they reach an age where they have access to linguistic cues about disjunction and its consequences [18]. It is not yet clear precisely how language helps—possibly by allowing the child to acquire an appropriate inferential structure, as we saw with number concepts.

Within a thinker, linguistic labels may be realized in a sensorimotor format ([2], pp. 15–17). If abstract concepts draw on linguistic labels, then a sensorimotor account of internal language use is good news for sensorimotor or embodied theories of abstract concepts. But note that this does not make for a solution to our grounding problem. Neither perception of speech sounds nor activation of the corresponding effectors serves to establish the reference of a concept—these resources do not serve to establish reference to fear as opposed to anger, for example. Sensorimotor patterns are distinctive of the words but are arbitrary with respect to meanings. They are not a resource that will tell us why anger is about *anger*, and fear *fear*.

Words also carry distributional data: about other words that tend to occur in the same context. Distributional data on its own is unstructured, making it hard, for example, to differentiate between anonyms, which tend to occur in the same contexts (e.g. hot/cold). However, in the case of natural number concepts discussed above, thinkers were not just using distributional information (e.g. ‘six’ and ‘seven’ are often heard together), but *structured* information: ‘six’ tends to occur after ‘five’ and before ‘seven’. Wiemer-Hastings & Xu [9] similarly emphasized the prevalence of structure in their production data on abstract concepts. hope is not simply a grab bag of happen, possible and want. A hope is something you want to happen. Here too the structure of sentences can act as a leg-up to getting the right structure between concepts. This is like another case of borrowed structure: where the structure of space is used to give thinkers a structure for their concepts of time [19].

So linguistic labels can be a means by which thinkers learn and implement a structure over some concepts. That structure is part of what grounds different concepts in different distinctive characteristics. Other sources of grounding remain important, even when language is playing a role. For example, mental state concepts probably draw on the mentalizing system, just as numerical concepts draw on the analogue magnitude system [20].

The picture I have presented so far expands well beyond the sensorimotor, grounding abstract concepts in other domain-specific systems like those dealing with numerosity, affect and mental states, all potentially structured by connections given to us in language. That is a rich picture indeed, but I want to argue that we will need to recognize more in order to account for grounding across the full range of abstract concepts.

## 4. Linguistic labels and deference

Language can help to ground concepts in ways that are internal or external to the thinker. The last section looked at the internal. This section looks at the external. Being linked to a public language word allows a concept to depend on a much wider body of knowledge than that which is stored by an individual concept user. For example, I may be quite unable to distinguish elms from beeches when out for a walk in the woods. Beyond my means of perceptual identification, various other pieces of information are encoded with my elm concept, e.g. that they are trees, with leaves, which they drop in autumn, etc. But again none of that information need be distinctive. Indeed, out of all the information stored with my elm concept, it could be that the only characteristic to distinguish it from beech is that elms are not beeches, and vice versa.

Nevertheless, the words ‘elm’ and ‘beech’ enable me to depend on the rich body of knowledge possessed by others in my linguistic community. I could learn, for instance, that deer are to be found in beech woods, and then rely on an expert to identify beeches when we are out in the woods looking for deer. The word associated with my beech concept allows me to enrich my own conception with information garnered from others. It also gives me new ways of using the concept without enriching my own conception, as when I rely on a friend to identify beeches. If I am using the concept in social contexts, then I do not need to internalize all the information shared by others. I can rely on the set of conceptions that are distributed among my linguistic community [21].

Usually, this socially mediated way of using concepts involves *deference* [22]. By deferring to experts, my elm and beech concepts are connected to distinctive characteristics encoded by them. Deference about how to distinguish elms from beeches is one source of grounding.^2^ If I do have views about what distinguishes elms from beeches, for example, having internalized some prototypical silhouette that makes me apply my beech concept, I hold those views tentatively. I stand to be corrected. I revise my conception when faced with someone who knows better. I may also defer about other facts about elms, beyond those used to pick them out or distinguish them, e.g. what to do with elm wood, how to cut it or use it. These are further plausibly distinctive grounding characteristics.

Linguistic labels combined with deference are good distinctive characteristics for grounding abstract concepts. Even with emotion concepts, where there are already rich internal resources as we have seen, linguistic deference may be responsible for grounding some fine-grained differences. For example, my shame and guilt concepts are certainly different, and I remember once being persuaded of what the differences are and why they are important. Not being an emotion specialist (in philosophy or normal life), I have forgotten, and there may be nothing else in the information stored internally with my two concepts to make them distinctive of one another. But the body of knowledge encoded by others in my linguistic community does distinguish between them, and public language words key my concepts into this knowledge, so that information encoded by others can act as distinctive grounding characteristics for my concepts. It is information encoded by others that makes my shame concept refer to a particular type of emotional state (i.e. to *shame*, not *guilt*).

It is particularly plausible for many social categories that deference is a key part of concept grounding. Consider these concepts about different ways we treat one another in social groups: marginalize, discriminate, segregate. They have many of the same connotations. Inexpert users may confuse them. But they pick out three different ways that we can treat people badly. Different words mark these differences, and enable users to learn the differences and become more proficient. In cases like these, concept users initially have little that makes the concepts distinct other than the associated words, and perhaps some vague differences in connotation and distributional properties. Users initially defer to others, learn more about the differences, and thereby acquire internally stored information that serves to distinguish between the concepts. Words act initially as distinctive characteristics based on deference to information in the linguistic community. They are then a route to individually stored distinctive characteristics.

For some social categories, we may almost all rely on deference to experts. I know what my friend in the pub promised, but am I right to categorize this under the concept contract? I know roughly what copyright is, but the conceptions stored in my copyright concept do little to make it distinctive from patent. Even with more everyday concepts like manslaughter, a certain amount of deference to legal experts is called for.

Deference is not automatic. For some concepts, people prefer their own judgements to those in the wider community. In identifying basic emotions like happiness and disappointment, people seem to prefer their own judgements as to how to classify faces, ignoring advice as to which cues are most reliable indicators of emotional valence [24]. Deference is a psychological attitude, carried by some concepts and not others.

Sometimes deference will be explicit. The concept user will say or think, ‘I don't know the difference between elms and beeches, I'd better ask someone’. This does not imply they think the category is ill-defined. We have plenty of evidence that copyright is a robust, reliable and widely used category; but most are willing to defer to experts as to when copyright arises and what the consequences are. At other times deference will be implicit. The concept user does not need to formulate a thought about deferring to others or make a reasoned decision. They may simply be inclined to trust others about how to apply the concept and inclined to relinquish their own conceptions in the face of testimony from those with more expertise.

Now for the point I have been building up to: deference is often metacognitive. Explicit deference is a belief about the thinker's own concepts. The thinker is making a judgement about their own mental states. For example, they are judging that their individual means for applying their elm concept are inadequate and that they should depend on others. Another kind of explicit deference concerns the word: ‘I don't know exactly what the word ‘marginalize’ means, so I'll defer’. This is also metacognitive. It is a belief about knowledge-of-meaning of the word. This metacognitive belief about the word will also serve to make the associated concept work deferentially. Its distinctive grounding characteristics can include characteristics encoded by others using the same word.

In cases where the beliefs driving deference are explicit, it is relatively straightforward to see when they are metacognitive. Deference can also be implicit, and those cases are harder to assess. Joëlle Proust distinguishes between analytic and procedural forms of metacognition [25,26]. Explicit deference is a case of analytic metacognition. Implicit deference is a candidate to be a form of procedural metacognition.

In procedural metacognition, a thinker selects, monitors and controls a cognitive activity without having thoughts about that activity. Simply carrying out a cognitive activity is not the same as monitoring and controlling it. So implicit deference need not be metacognitive. It can just be part of the normal object-level activity of encoding new information about a subject matter. When I hear from a colleague that Tasmanian devils have an infectious form of cancer, I may encode that information just because it seems more probable than not. But some forms of implicit deference can be metacognitive. Deference may be driven by a disposition or feeling that operates in a unified way across all the information encoded in a concept, making all the information susceptible to revision based on testimony, irrespective of the probabilistic strength of the beliefs encoded. This could be present for some concepts and absent for others, so that for concepts otherwise matched in the type and strength of information stored, some would be very malleable in the face of testimony, others not. It is a harder distinction to draw in the implicit case, and it will turn on empirical details about the precise functional role of deference in a thinker's concept-forming practices, but it is at least plausible that some forms of implicit deference fall into Proust's category of procedural metacognition.

Deference can mean that a category itself is socially constructed, but need not. Where a category is socially constructed, people's dispositions to classify things partly determine what falls into the category and what does not. Legal terms are a clear case. Application of legal concepts like murder and manslaughter is fixed by legislation, interpreted and modified by the courts. Ultimately, whether a killing counts as a case of manslaughter depends on how the courts classify it. This does not mean the category has no inductive potential. Very many features differ between a manslaughter and an accidental killing, and, of course, the consequences for the perpetrator are very different.

Often social processes produce categories with as much inductive power as paradigmatic natural kinds. Consider the category of monks. When you can categorize someone under the concept monk, that tells you a lot about their way of life and socio-economic status. Within a particular culture, it might even allow you to guess something about age, accent and dress (consider monks in Tibet). But that is partly because how people dress and behave is influenced by their having been classified under the concept, i.e. as a result of being ordained. Our classificatory processes have a causal role in forming the cluster of properties that members of the category tend to share [27]. These categories, therefore, depend partly on human minds, but it would be wrong to think of them as existing only in the mind. Monks tend to share various mind-independent properties (e.g. attire) and the fact that those properties tend to cluster together is a real feature of the world. Minds are causally involved in creating the cluster, but the resulting dimensions of similarity are not mind-dependent. Deference opens the door to many abstract concepts of categories that are socially constructed, but we should not think the categories are as a result any less important, or any less real features of things in the world (e.g. of people).

Finally, I ask whether the list of potentially grounding attributes referred to at the outset (cf. introduction to this theme issue) covers metacognitive deference. Introspection was on the list. Does that cover metacognitive deference? Introspection is something more than being in a mental state or having a subjective experience. It is a matter of reflecting on or self-ascribing that mental state or experience. Explicit metacognitive deference seems to fall within this definition, but implicit metacognitive deference need not. So we need to enlarge this list somewhat to ensure it extends to procedural metacognition. The list of potentially distinctive grounding characteristics should extend to both explicit and implicit metacognition.

## 5. Metacognition about concepts

Deference is one metacognitive feature that looks to be involved in grounding some abstract concepts. I want to close by suggesting another one that has been little remarked-on previously. Many concepts are constructed, not by deference to experts, but by a collective process of deciding how to use a concept. We also decide collectively to abandon some concepts and to adopt others. I want to argue that metacognitive assessments are often involved in the social processes through which concepts come into and go out of common use.

In academic research, this is often a self-conscious pursuit. There has been a long debate about whether the concept innate is helpful, or whether it should be abandoned [28]. Within psychology, the idea of repressed memory is much more contested than working memory. The physical concept attach seems much more secure than the psychological concept social attachment. When we discuss our theoretical repertoire in this way, we express explicit views about how good or bad the concepts are for scientific purposes. That is a bit of explicit metacognition: an explicit judgement about a concept. A similar process operates implicitly, as when people use a tone of voice or scare quotes to distance themselves from a concept (e.g. by those reluctant to use the concept innate).

Social epistemology has begun to examine how knowledge-forming practices are often collective [29]. Constructing the concepts that encode our knowledge is no less a collective endeavour. One of the key forms of sharing between agents, which makes two heads better than one for performing some tasks, is metacognitive: communication about one's own confidence or reliability [30]. Indeed, it is plausible that the reason we have explicit access to the reliability of our mental processes—the reason facts about our cognitive processes are made conscious and available for the verbal report—is to allow us to share reports of confidence and other metacognitive parameters in the service of working together [31].

We can see the social processes of concept construction at work in the history of science. Thomas Kuhn described periods of scientific revolution where an accumulation of problems and anomalies leads to one conceptual scheme being overthrown and replaced by a new set of concepts [32]. A paradigm example is the replacement of Newtonian physics with relativity and quantum mechanics. The concept space–time was added and ether jettisoned. The fact that the process of *concept* construction is social does not, in fact, imply that the *category* referred to by the concept must be socially constructed (although that separation can be disputed). Neither space–time nor the molecular gene depends for their existence on minds or our classificatory practice. But the social processes of science explain how it is that we now have the concepts space–time and molecular gene.

For the social concepts human rights and democracy, for example, social concept construction goes hand-in-hand with social construction of the category. Not only do we collectively decide that the concept human rights is a good one to use, but we also decide collectively what the ambit of the concept should be. (The latter process is especially complex, and it is hard to know in many cases what makes us home in on a particular usage.) Examples include citizenship, emancipation and accountability. Concepts judged as useful can also be negatively valenced: discrimination, inequality, climate change. In other cases, we disagree about whether the phenomenon they pick out is good or bad: monarchy, capitalism, charity. Note that we have this debate while implicitly agreeing that these are good ways of describing the world—the concept imperialism is useful precisely because it allows us to decry instances when we see them. We negotiate the boundaries of a concept, and we can also debate whether a particular concept should be used, replaced or abandoned altogether.

Sometimes it is difficult to assess whether a debate is about a concept or about the category it refers to. Plastic is more versatile and useful than jute—that is clearly a judgement about the category. How about expressing a preference for centimetres over inches? Is that primarily a preference for one concept over another, or is it preference between two length properties? Social processes of concept selection need not involve explicit judgements and then the distinction may be unclear. However, there are cases where a social debate clearly is about a concept and whether we should use it. Racial concepts are like that. We have an ongoing debate about whether we ought to be categorizing people in racial terms [33]. So long as many people are still disposed to make racial categorizations, other things may flow from being put in a racial category (e.g. rough generalizations about socio-economic status). For this reason, the racial concepts may not be devoid of inductive power. But we can still form the view that we ought not to think in racial terms—we ought not to use the racial concepts; and we might in time decide collectively to stop using them.

Social categories are a common source of controversy and so social concepts are a good place to look to see metacognitive assessments of our concepts at work. The scientific examples show that a similar social process is likely to be at work there too. How is this relevant to the distinctive grounding characteristics problem? The answer is still deference: the phenomenon of deference in concept use, explicit and implicit, means that the characteristics of a concept that feed into the metaphysical account of reference determination need not all be found in the mind of the individual concept user. This section has argued that deference is not simply deference to experts, but can be deference to a wider social process in which no individual is an expert; and also that other forms of metacognition about concepts are involved in this process. Metacognition helps to guide the collective choice to embrace some concepts and jettison others. These are two ways that metacognition is important for solving the distinctive grounding characteristics problem for abstract concepts. While not confined to abstract concepts, the connection to a wider group of concept users is especially important for abstract concepts, which by definition lack sufficiently distinctive sensorimotor characteristics to act as distinctive grounds by themselves.

## 6. Conclusion

To see how abstract concepts are grounded in characteristics that make them distinctive from one another and allow them to refer to different properties, we need to go well beyond the sensorimotor, encompassing other domain-specific systems like affect, mentalizing and tracking analogue magnitudes. For many abstract concepts, we need to go further still and appeal to linguistic labels as a distinguishing resource, either because labels give the thinker a structure over a set of concepts, or because they allow the thinker to defer to information in their linguistic community. Deference in using a concept, together with explicit and implicit assessments of its dependability, are metacognitive processes that regulate concept use. Thus, metacognition about concepts is a hitherto missing ingredient that should be added to the list of resources needed if the distinctive grounding characteristics problem is to be solved for the whole range of abstract concepts.

## Endnotes

1 I use small capitals when naming concepts, and sometimes italics to emphasize when I am talking about a property; here the property an action, event or relation has when it is *fair*.

2 People seem to assume that they have direct access to distinctive characteristics for which they in fact need to draw on an expert [23], but that illusion is not required for the characteristic encoded by the expert to play the role of a distinctive grounding characteristic.

## Data accessibility

This article has no additional data.
